# Suppression of HTLV-1 transcription by SIRT1 deacetylase

**DOI:** 10.1186/1742-4690-12-S1-P53

**Published:** 2015-08-28

**Authors:** Dong-Yan Jin, Hei-Man Vincent Tang, Wei-Wei Gao, Chi-Ping Chan, Hidekatsu Iha, Kit-San Yuen

**Affiliations:** 1 Department of Biochemistry, University of Hong Kong, 21 Sassoon Road, Pokfulam, Hong Kong; 2 Department of Infectious Diseases, Oita University, Yufu, Japan

## 

Infection with HTLV-1 causes adult T-cell leukemia and tropical spastic paraparesis in different subsets of infected people. Treatments for HTLV-1-associated diseases are unspecific and unsatisfactory. Prophylactic measures have not been developed. Although HTLV-1 pathogenesis involves multiple stages and factors, high proviral load has been singled out as a major risk factor which predicts disease. HTLV-1 encodes Tax transactivator that potently activates transcription from viral long terminal repeats (LTR) and cellular promoters harbouring cAMP-responsive or KB element. Blocking Tax activity in infected cells should provide prophylactic and therapeutic benefits. In this study, we characterize the negative regulatory role of SIRT1 deacetylase in Tax-induced activation of HTLV-1 LTR. SIRT1 is a sirtuin with anti-cancer and anti-aging activity. Whereas over expression of SIRT1 abolished the activity of Tax to activate the LTR, compromising SIRT1 by RNA interference augmented Tax activation of the LTR. Resveratrol is a natural inhibitor of SIRT1 widely sold as a nutritional supplement and extensively tested for beneficial effects in various diseases. A SIRT1-dependent inhibition of the transcriptional activity of Tax was observed in cells treated with resveratrol. Consistently, treatment of HTLV-1-transformed T cells with resveratrol led to the activation of SIRT1 and the suppression of HTLV-1 proviral transcription. On the contrary, specific inhibition of SIRT1 by Sirtinol or Ex527 promoted HTLV-1 mRNA expression. The amount of HTLV-1 virion collected from culture supernatant was decreased in MT2 cells treated with resveratrol. Tax was found to interact with SIRT1 in HTLV-1-transformed T cells. Administration of resveratrol blocked the interaction of Tax with CREB and suppressed the recruitment of CREB and CRTC1 to the LTR. Taken together, our findings document the suppression of Tax activation of HTLV-1 transcription by SIRT1 and reveal potential benefits of small-molecule activators of SIRT1 such as resveratrol in the prevention and intervention of HTLV-1-associated diseases. Supported by SKY-MRF (2011), HK-RGC (HKU7674/12M, HKU7686/13M and HKU1/CRF/11G) and HK-HMRF (13121052) grants.

